# Static magnetic field-modulated mesenchymal stem cell-derived mitochondria-containing microvesicles for enhanced intervertebral disc degeneration therapy

**DOI:** 10.1186/s12951-024-02728-6

**Published:** 2024-07-31

**Authors:** Pengzhi Shi, Haiyang Gao, Zhangrong Cheng, Kangcheng Zhao, Yuhang Chen, Xianglong Chen, Weikang Gan, Anran Zhang, Cao Yang, Yukun Zhang

**Affiliations:** Department of Orthopedics, Union Hospital, Tongji Medical College, Huazhong University of Science and Technology, Wuhan, 430022 China

**Keywords:** Mesenchymal stem cell, Mitochondrial, Microvesicle, Intervertebral disc degeneration, Static magnetic field

## Abstract

Intervertebral disc degeneration (IVDD) is characterized by the senescence and declining vitality of nucleus pulposus cells (NPCs), often driven by mitochondrial dysfunction. This study elucidates that mesenchymal stem cells (MSCs) play a crucial role in attenuating NPC senescence by secreting mitochondria-containing microvesicles (mitoMVs). Moreover, it demonstrates that static magnetic fields (SMF) enhance the secretion of mitoMVs by MSCs. By distinguishing mitoMV generation from exosomes, this study shifts focus to understanding the molecular mechanisms of SMF intervention, emphasizing cargo transport and plasma membrane budding processes, with RNA sequencing indicating the potential involvement of the microtubule-based transport protein Kif5b. The study further confirms the interaction between Rab22a and Kif5b, revealing Rab22a’s role in sorting mitoMVs into microvesicles (MVs) and potentially mediating subsequent plasma membrane budding. Subsequent construction of a gelatin methacrylate (GelMA) hydrogel delivery system further addresses the challenges of in vivo application and verifies the substantial potential of mitoMVs in delaying IVDD. This research not only sheds light on the molecular intricacies of SMF-enhanced mitoMV secretion but also provides innovative perspectives for future IVDD therapeutic strategies.

**Supplementary Information:**

The online version contains supplementary material available at 10.1186/s12951-024-02728-6.

## Introduction

Low back pain (LBP) is a prevalent clinical manifestation of degenerative alterations within the musculoskeletal system, predominantly occurring in the middle to elderly age cohorts. Evidently, a substantial 80% of individuals experience LBP at some point in their lives [1, 2]. Despite the multifaceted etiology of LBP, identifying specific causative factors remains challenging [3]. Current scholarly discourse posits intervertebral disc degeneration (IVDD) as the principal cause of LBP [4]. The intricate intervertebral disc (IVD) structure, comprising the central nucleus pulposus (NP), peripheral annulus fibrosus, and cartilaginous endplate interposed between superior and inferior vertebrae, underscores that disruption in the physiological function of any component may destabilize the IVD, precipitating disc degeneration. In current clinical practice, the primary treatment options for IVDD-related diseases include conservative therapy and surgical intervention. However, neither approach can fundamentally reverse the degeneration of the IVD. In particular, surgical treatment, while capable of alleviating symptoms to some extent, fails to restore the normal height of the disc and carries the risk of accelerating the degeneration of adjacent vertebral segments [5].

The pivotal buffering role of the central NP is underscored by its synthesis and secretion of proteoglycans within the extracellular matrix, crucial for maintaining IVD homeostasis. Under adverse conditions such as heightened loads and inflammatory environments, nucleus pulposus cells (NPCs) frequently undergo mitochondrial dysfunction, resulting in energy insufficiency, cellular senescence, and apoptosis. This leads to impaired cell proliferation and dysregulated synthesis and metabolism of the extracellular matrix (ECM). These changes are considered a significant hallmark in the pathogenesis and progression of IVDD, ultimately leading to alterations in spinal biomechanics, causing LBP and even disability [6]. Consequently, augmenting the activity and energy provision for NPCs is a crucial strategy for mitigating IVDD.

Intercellular communication is a pivotal process facilitating the transmission of communication mediators from one cell to another, thereby playing a crucial role in the maintenance of dynamic tissue or organ equilibrium [7]. Diverse communication mediators, including proteins, nucleic acids and lipids, actively participate in orchestrating intercellular communication [8]. Recent research has unveiled that certain cellular organelle, notably mitochondria, can also serve as communication mediators in the context of intercellular communication [9]. Mitochondrial transfer between cells emerges as a dual-faceted mechanism, serving both to eliminate dysfunctional mitochondria and as a rescue mechanism responding to environmental stressors. Mechanistically, intercellular mitochondrial transfer manifests through three principal modalities: (1) the formation of transient cell connections, such as tunneling nanotubes (TNT), enabling the movement of mitochondria from one cell to another; (2) the extrusion of mitochondria within extracellular vesicles (EVs), facilitating their delivery to recipient cells; and (3) the release of free mitochondria for subsequent capture by recipient cells [10–13].

EVs, characterized by lipid bilayer nanovesicles of cellular origin, play a crucial role as indispensable mediators in intercellular communication, exhibiting a ubiquitous presence across diverse tissues and bodily fluids [14]. These vesicles serve as conduits for the transport of a myriad of bioactive cargoes, thereby modulating receptor signaling and manifesting diverse biological effects [15, 16]. Notably, accumulating evidence supports the preferential enrichment of distinct mitochondrial contents within specific subpopulations of EVs [17]. Among these, smaller EVs such as exosomes are documented to predominantly carry small RNAs and mitochondrial DNA (mtDNA) [18, 19]. In contrast, larger EVs, exemplified by microvesicles (MVs), have been identified as potential carriers of entire mitochondria [20]. Moreover, EVs harboring mitochondrial cargo possess the capability to convey mitochondrial components, thereby influencing the metabolic milieu and phenotypic characteristics of recipient cells [21]. Studies delineate that mitochondria-containing EVs originating from healthy cells facilitate the delivery of functional mitochondrial fragments to recipient cells, thereby contributing to the restoration of mitochondrial biogenesis and energy metabolism [22].

Unlike the biogenesis mechanism of exosomes, MVs primarily emerge through the outward budding and fission of the plasma membrane, mediated by the contraction of the actin cytoskeleton, exhibiting diameters ranging from 100 nm to 1 μm [23]. During the budding process from the plasma membrane, cargo initially assembles on the cytoplasmic surface and subsequently differentiated membrane microdomains, indicative of outward budding, emerge on the cell surface. Subsequently, vesicle fission occurs, leading to the rapid release of vesicles into the extracellular space [24]. Nonetheless, the precise mechanisms underpinning the genesis of mitochondria-containing MVs and the intravesicular transport of mitochondrial components remain elusive.

**Scheme. 1:**
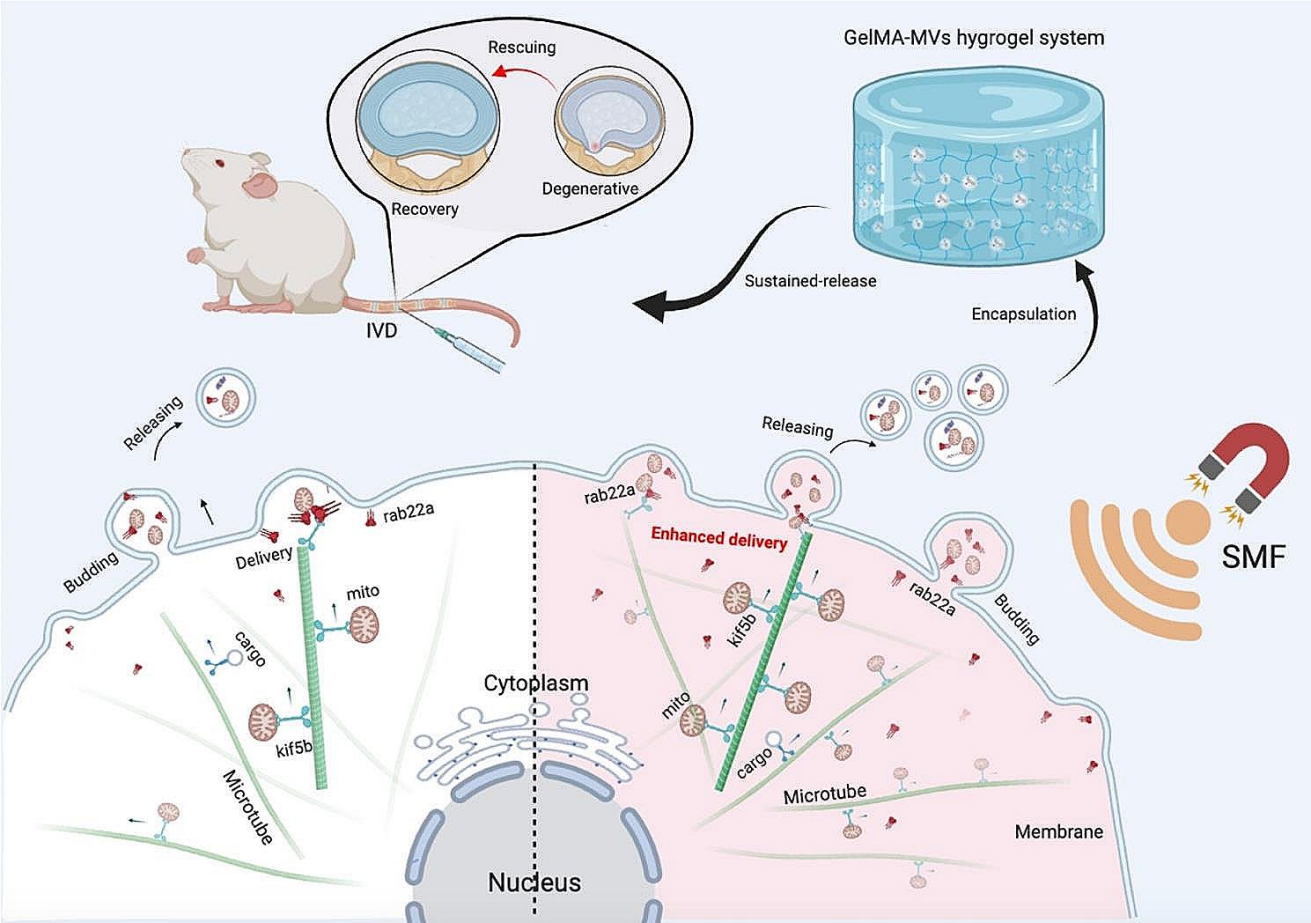
The static magnetic field (SMF) enhances the mitochondrial transport capacity of Kif5b and promotes the interaction between Kif5b and Rab22a, facilitating the sorting of mitochondria into microvesicles (MV) derived from mesenchymal stem cells (MSCs) and membrane budding. MitoMV obtained after SMF intervention, when encapsulated in hydrogels, demonstrates significant therapeutic efficacy in the treatment of intervertebral disc degeneration (IVDD)

Empirical evidence underscores a consequential correlation between the quantity and cargo composition of MVs and the intricacies of the cellular microenvironment [25, 26]. The cellular response to stressors emerges as a determinant factor, influencing both cell morphology and the biophysical properties of the plasma membrane, inclusive of alterations in secretion dynamics. In turn, this response augments intercellular communication and fortifies regenerative capacities [27]. Static magnetic field (SMF) have a modulatory effect on cellular entities by manipulating their orientation and polarity [25, 28]. Furthermore, the impact of SMFs extends to the reorientation of macromolecules, thereby intricately influencing the polarity and spatial distribution of cellular organelles [29]. Noteworthy is the observed directional release of various bioactive factors by cells, such as membrane-derived vesicles or exosomes. In the context of this study, the investigation commenced by exploring the secretion of mitochondria-containing MVs (mitoMVs) by mesenchymal stem cells (MSCs) as a potential intervention to decelerate IVDD. Subsequent scrutiny revealed that SMF intervention amplifies the secretion of mitoMVs by MSCs (Scheme 1). Consequent to this observation, a detailed analysis was undertaken to delineate the nuanced mechanisms governing the secretion of mitoMVs by MSCs following SMF intervention. To overcome the limitations intrinsic to in vivo mitoMVs therapy, particularly the abrupt release associated with local injection into the IVD, this study ingeniously devised a photocurable hydrogel delivery system. This system serves to encapsulate mitoMVs, thereby orchestrating a sustained and controlled release, culminating in an enhanced therapeutic strategy for IVDD.

## Materials and methods

### Human nucleus pulposus (HNP) tissue samples

Clinical samples of HNP tissue were obtained from patients who underwent spinal fusion due to degenerative diseases of the lumbar spine. Informed consent of this study was obtained from all the participants. Magnetic resonance images (MRI) degree of disc degeneration was evaluated according to the Pfirrmann grading system. Ethical approval was obtained from the Ethics Committee of Tongji Medical College, Huazhong University of Science and Technology.

### Isolation and culture of NPCs and MSCs

This animal experiment was approved by the Animal Ethics Committee of Wuhan Myhalic Biotechnology. Sprague Dawley rats (2–3 months) were purchased from the Laboratory Animal Center of Wuhan Myhalic Biotechnology (Wuhan, China). NPCs and MSCs were separately harvested from the coccygeal IVD tissues and femoral bone marrow cavity when rats were euthanized by overdose of 2% (w/v) pentobarbital. Then, the NP tissues were isolated under a dissecting microscope and digested in 0.2% type II collagenase (Gibco, USA) for 12 h at 37 ℃ with 5% CO_2_. The obtained cells and partially digested tissues were washed with phosphate-buffered saline (PBS) twice and centrifuged at 1000 rpm for 5 min, and then were cultured in Dulbecco’s modified Eagle’s medium supplemented with the F12 nutrient mixture and 15% fetal bovine serum (Gibco, 10,099,141 C) at 37 °C with 5% CO2. The obtained bone marrow blood was cultured in MSC complete medium (Cyagen, USA) at 37 ℃ with 5% CO_2_. Then, the culture medium was changed every three days. The cells were passaged at a 1: 3 ratios at 80–90% confluence, and cells from the passage 2–3 were used in further experiments. The senescent cell model was established by exposing NPCs to tert-butyl hydroperoxide (TBHP, 100 µM; Sigma-Aldrich, A13926) for 12 h followed by replacing with normal medium in vitro experiments.

### Magnetic field exposure and cell viability assay

The SMF was generated by rectangular Nd-Fe-B magnets of 50*30*5 mm placed under each culture plate. A gaussmeter (SanLiang, Japan) is used to measure the magnetic field strength on the surface of the magnets at different distances. The distance between the cell culture plate and the magnet surface was adjusted to maintain a fixed range of magnetic field strength.

MSCs were seeded in 96-well plates at a density of 1 × 10^4 cells per well and were incubated in complete medium overnight at 37 ℃ with 5% CO_2_. Then, MSC were treated with different magnetic field strength. Cell counting kit-8 (CCK-8, Dojindo, Japan) was added into each well and incubation for 1 h, the optical density value (OD) was read at 450 nm by Microplate Reader (Bio-Rad, Hercules, USA).

### Cell proliferation and live/dead assay

MSCs were seeded in cell slide of 12-well plate and treated with different magnetic field strength. EdU Cell Proliferation Kit (Beyotime, China) was used to detect the proliferation of MSCs. Subsequently, cells were incubated with EdU for 2 h and fixed with 4% paraformaldehyde for 15 min, and then were incubated with 0.3% Triton X-100 for 10 min according to the manufacturer’s instructions. Then, cells were incubated with Click Reaction Mixture for 30 min and then incubated with Hoechst 33,342 for 10 min in a dark place. At last, MSCs were observed and recorded using fluorescence microscope and analyzed by Image J software.

For live/dead assays, the NPCs were co-culture on the surface of GelMA hydrogels at 37 ℃ with 5% CO_2_. Then 100 µL of live/ dead staining solution (Beyotime, China) was added into cell plate. Subsequently, NPCs were incubated in darkness at 37 ℃ for 30 min. At last, NPCs were observed and recorded using fluorescence microscope and analyzed by Image J software.

### Transwell co-culture system

To assess the impact of MSCs on senescent NPCs, a transwell co-culture system was constructed (ratio of MSCs and NPCs 1:1). In brief, NPCs were seeded in a 6-well plate and subjected to 100 µM TBHP incubation to induce senescence. Transwell (8 μm, 0.1 μm, 6 wells, BIOFIL, China) were placed in another 6-well plate, and MSCs were seeded in transwell. Then, transwell seeded MSCs was transferred to the corresponding well containing NPCs to establish the co-culture system.

For the assessment of mitochondrial horizontal transfer, NPCs and MSCs were separately labeled with mito-Tracker Red CMXRos (100 nM, Beyotime, China) and mito-Tracker Green (100 nM, Beyotime, China) after seeding. The co-culture was subsequently initiated to allow for the examination of mitochondrial dynamics and transfer between the two cell populations.

### Senescence β-galactosidase staining

NPCs were seeded in a cell slide of 12-well plate and treated with TBHP (100 µM) for 12 h. Senescence β-Galactosidase (SA-β-Gal) staining was performed according to the manufacturer’s instructions from the SA-β-Gal staining Kit (Beyotime, China). NPCs were observed using inverted phase contrast microscope and were analyzed by Image J software.

### JC-1 assay for mitochondrial membrane potential

Mitochondrial membrane potential (MMP) was measured using the JC-1 (5,5’,6,6’-tetrachloro-1,1’,3,3’-tetraethylbenzimidazolcarbocyanine iodide) Detection Kit (Beyotime, China). NPCs from different interventions were washed with PBS and incubated with 2 µM JC-1 dye for 20 min. Then, the cells were washed with incubation buffer two times and analyzed using flow cytometry.

### Reactive oxygen species (ROS) measurement

The level of intracellular ROS was measured by DCFH-DA (MCE, China) of cell-permeable probe. After different interventions in a 12-well plate, NPCs were washed twice with PBS and incubated with 20 µM DCFH-DA for 1 h at 37 ℃ according to the manufacturer’s instructions. Then, the cells were harvested and analyzed using flow cytometry.

### ATP measurement

The level of intracellular ATP was measured by ATP Assay Kit (Beyotime, China) according to the manufacture’s protocol. Briefly, NPCs were lysed using lysis buffer and centrifuged at 12,000×g for 5 min at 4 °C after different interventions in a 6-well plate. The supernatants were mixed with ATP detection buffer containing luciferase. Luminance (RLU) was analyzed by using a multifunctional enzyme marker (PerkinElmer, USA). The concentration of ATP was calculated according to standard curve and normalized using the cellular protein level.

### Isolation and characterization of MVs

To isolate MVs in vitro, MSCs were cultured in fresh medium (10% exosome-depleted FBS, Gibco, USA). The conditioned medium was harvested and centrifuged at 300×g for 10 min to pellet non-adherent cells. The supernatant was then subjected to a centrifugation step at 2,000×g for 20 min to pellet apoptotic bodies and cell debris. Subsequently, the supernatant was further centrifuged at 20,000×g for 60 min to pellet MVs. The total protein content in MVs was quantified using BCA assay kit (Beyotime, China). The particle concentration and size of MVs were measured by Flow NanoAnalyzer (Nanofcm, China). Transmission electron microscopy (TEM, Hitachi, Japan) was used to measure morphologies and contents of MVs. The MV dose applied in the cell experiment was 1 × 10^9/mL.

To isolation of labeling MVs, MSCs were incubated with Mito-Tracker (100 nM, 30 min) and DiO (10 µM 20 min, Beyotime, China), or SMF intervention at 37 ℃ followed by replacing with normal medium for 24 h. Then, the conditioned medium was harvested and centrifuged according to above protocol. The flow cytometry assays were utilized to identify the subsets of MVs.

### RNA sequencing (RNA-seq) analysis

RNA-seq was performed by SeqHealth Biotechnology (Wuhan, China). Briefly, Six RNA samples were isolated using TRIzol reagent (Invitrogen, USA) and quantified using NanoDrop 2000 (ThermoFisher, USA). cDNA library was construction and followed by sequencing was performed by using Illumina Novaseq platform. Clean data were obtained by removing reads containing adapters, poly-N, and low-quality reads from the raw data. Transcripts were reconstructed using StringTie, and clean data were mapped to the reference genome of Rattus norvegicus. Gene expression was analyzed based on fragments per kilobase of transcript per million mapped fragments (FPKM). Differential gene expression analysis was conducted using the DESeq R package, with adjusted p-values (FDR < 0.05) and |log2foldchange| (|FPKM|> 0.58) considered as thresholds for significant differential expression. Finally, the enrichment analysis of differentially expressed genes were performed by clusterProfiler of R package in R software.

### Small interfering RNA (siRNA) knockdown

Cells were cultured in the 6-well plates and transfected with siRNA-Kif5b (si-Kif5b) and siRNA-Rab22a (si-Rab22a) (Tsingke, China) using a transfection kit (C10511-1, RiboBio, China) according to the manufacturer’s instructions. The siRNA sequences were listed in Table S1.

### Co-immunoprecipitation (CO-IP) assay

To verify the protein-protein interactions, we performed CO-IP using specific antibodies and protein A/G beads (MCE, China). Briefly, we collected the protein samples from MSCs after intervention and lysed them with RIPA buffer containing protease and phosphatase inhibitors. Then, MSCs protein lysates were coimmunoprecipitated with an anti-Kif5b antibody or isotype antibody with protein A/G beads at 4 °C overnight on a rotator. After washing with NP-40 lysis buffer twice, the proteins samples were separated from beads with 5×SDS loading buffer at 95 °C for 10 min and analyzed with a Coomassie blue staining and Western blot assay.

### Synthesis of GelMA-MV hydrogel

To prepare GelMA hydrogel, we dissolved gelatin (type A, 300 bloom) in PBS with a concentration of 10% (w/v), followed by adding methacrylic anhydride (MA) (0.8 mL/g of gelatin) dropwise to the gelatin solution under stirring at 50 °C for 3 h. NaOH (1 M) were used to adjusted the pH of the reaction mixture to 7.4. GelMA solution were dialyzed against deionized water for 7 days at 4 °C followed by lyophilizing the GelMA solution.

To achieve GelMA-MV hydrogel, we dissolved GelMA powder in PBS at 37 °C (10% w/v) and added obtained MVs (1 × 10^10^/mL) to obtain GelMA-MVs solutions. Lithium phenyl-2,4,6-trimethylbenzoylphosphinate (LAP, 0.05% w/v) were added to the GelMA-MVs solutions. Then, we exposed the GelMA solutions to UV light (365 nm, 10 mW/cm^2^) for 20s to induce photo-crosslinking.

### Characterization of GelMA-MV hydrogel

To identify the morphology of GelMA and GelMA-MVs hydrogel, we lyophilized the hydrogel using freeze dryer (Scientz-10 N) and observed the hydrogel under a scanning electron microscope (SEM, TESCAN MIRA LMS, Czekh). Then the pore diameter was measured.

To detect the degradability and swelling ratio of hydrogels, we considered the initial mass of hydrogels was W0. Then the hydrogels were immersed in 2 U/mL collagenase or PBS solution and incubation at 37 °C. The remaining mass and swelling mass were recorded over time as W1 and Wt. The mass remaining ratio and swelling ratio were calculated as follows: Mass remaining ratio (%) = W1/W0 × 100%. Swelling ratio (%) = (W1 − W0)/W0 × 100%.

To measure the ratio of MV released, the hydrogels containing DiR-labeled MVs were immersed in 2 U/mL collagenase solution and incubation at 37 °C. The fluorescence intensity was analyzed using multifunctional enzyme marker (ENVISION2105, PerkinElmer, USA). The ratio of MVs released was calculated by measuring the fluorescence intensity of the MVs released at the defined time points.

### Western blot (WB) assay

Total protein was extracted from cells by Whole Cell Lysis Assay (Keygen, China) and the protein concentration was measured by using the BCA protein assay kit (Beyotime, China, catalog no. P0010). Then, an equal protein sample of each group was subjected to the SDS-PAGE and transferred onto the PVDF membrane. After that, the membranes were blocked with 5% non-fat milk for 2 h at room temperature and then were incubated with primary antibodies against Kif5b, P16, P21, Annexin A, CD9, HSP60, VDAC, COX IV and GAPDH (Proteintech, China); β-tubulin and Rab22a (Zenbio, China), overnight at 4 ℃. After washing three times with TBST (Tris-buffered saline and 0.1% Tween 20), the membranes were incubated with secondary antibodies (Proteintech, China) for 2 h at room temperature. Then, the membranes were visualized using an enhanced chemiluminescence system. Finally, the relative amount of protein was analyzed using ImageJ software.

### IVDD animal model induction

Twenty Sprague-Dawley rats were divided into five groups randomly (*n* = 4 per group). Briefly, the rats were anesthetized by intraperitoneal injection of 0.5 mL/100 g 2% (w/v) pentobarbital (40 mg/kg). After sterilization with povidone iodine, coccygeal IVD (Co 6/7) were percutaneously punctured by a 21G needle at a depth of 5 mm, followed by rotation at 360° and holding for the 30s. After 2 weeks, the experiments groups were treated with 2 µL GelMA, GelMA-MVs and GelMA-mitoMVs (MVs isolated after 40 mT SMF intervention, 1 × 10^10/mL) respectively, while the PBS group were treated with equivalent volume of PBS once a week for 6 weeks. The DiR-labeled GelMA-MVs hydrogel system was imaged using In-Vivo Xtreme II small animal imaging system (Bruker Corporation, Billerica, MA) on days 0, 3, 7, 10 to assess the degradation of MVs in vivo.

### MRI evaluation

Radiographic and MRI scans were carried out 6 weeks after puncture. The rats were placed in a prone position after anesthetizing. The signal and structural change of the IVD were obtained by MRI scans (3.0T, GE, CT, and US). Briefly, SD rats were maintained after induction of anesthesia by inhalation of 2% isoflurane. Then, rats were placed in a prone position for examination. Sagittal T2-weighted images were evaluated according to the Pfirrmann grade [30]. The disc height index (DHI) was measured by Image J software [31].

### Histological and immunohistological analysis

The specimens were harvested and fixed with 4% paraformaldehyde, decalcified with 10% ethylenediaminetetraacetic acid solution, and embedded in paraffin. The specimens were cut into 5-µm sections, and the histological features were examined by hematoxylin and eosin (HE), safranin-O-fast green stains and alcian blue staining. Histologic images were evaluated following histologic grading scale criteria [32].

Tissue sections were incubated with the primary antibodies (P21 (1:500; Proteintech 10355-1-AP), P16 (1:200; Proteintech 10883-1-AP)) at 4 °C overnight. Then, the sections were washed with PBS and incubated with the secondary antibody for 60 min at room temperature (diluted 1:200; Proteintech, PR30011 for rabbit and PR30012 for mouse).

### Immunofluorescence analysis

For cell slide staining, MSCs at 60% confluence were exposed to different treatments. After incubation with mito-Tracker Red CMXRox, the cell slides were fixed with 4% paraformaldehyde at room temperature for 20 min. After permeabilization and blocking, co-staining of Kif5a and Rab22a of cell slides was performed using a Three-color Fluorescence kit (Recordbio Biological Technology, Shanghai, China) based on the tyramide signal amplification (TSA) technology according to the manufacture’s instruction. The nuclei were stained with DAPI (Beyotime, P0131-5 ml) and the slides were observed using a laser scanning confocal microscope (Olympus, BX53).

For tissue sections staining, the sections were fixed in 4% paraformaldehyde for 30 min and washed twice with PBS. After permeabilization and blocking, the sections incubated with primary antibodies against P16 (1:100; Proteintech, 10883-1-AP), P21 (1:100; Proteintech, 10355-1-AP) at 4 °C overnight. Then the sections were washed twice with PBS and incubated with CoraLite594-conjugated goat anti-rabbit IgG (1: 200, Proteintech, China) secondary antibodies for 1 h at room temperature in the dark. The sections were photographed by a fluorescence microscope and analyzed by Image J software.

### Statistical analysis

All of the data were analyzed by GraphPad Prism 9 software (GraphPad Software, USA) The quantitative data were expressed as mean ± standard deviation (SD). The data of multiple independent groups were analyzed by one-way ANOVA. Student’s t-test was used to analyze the differences between the two groups. P-value < 0.05 was considered to be significant.

## Results

### MSC co-culture delays NPC senescence

**Fig. 1:**
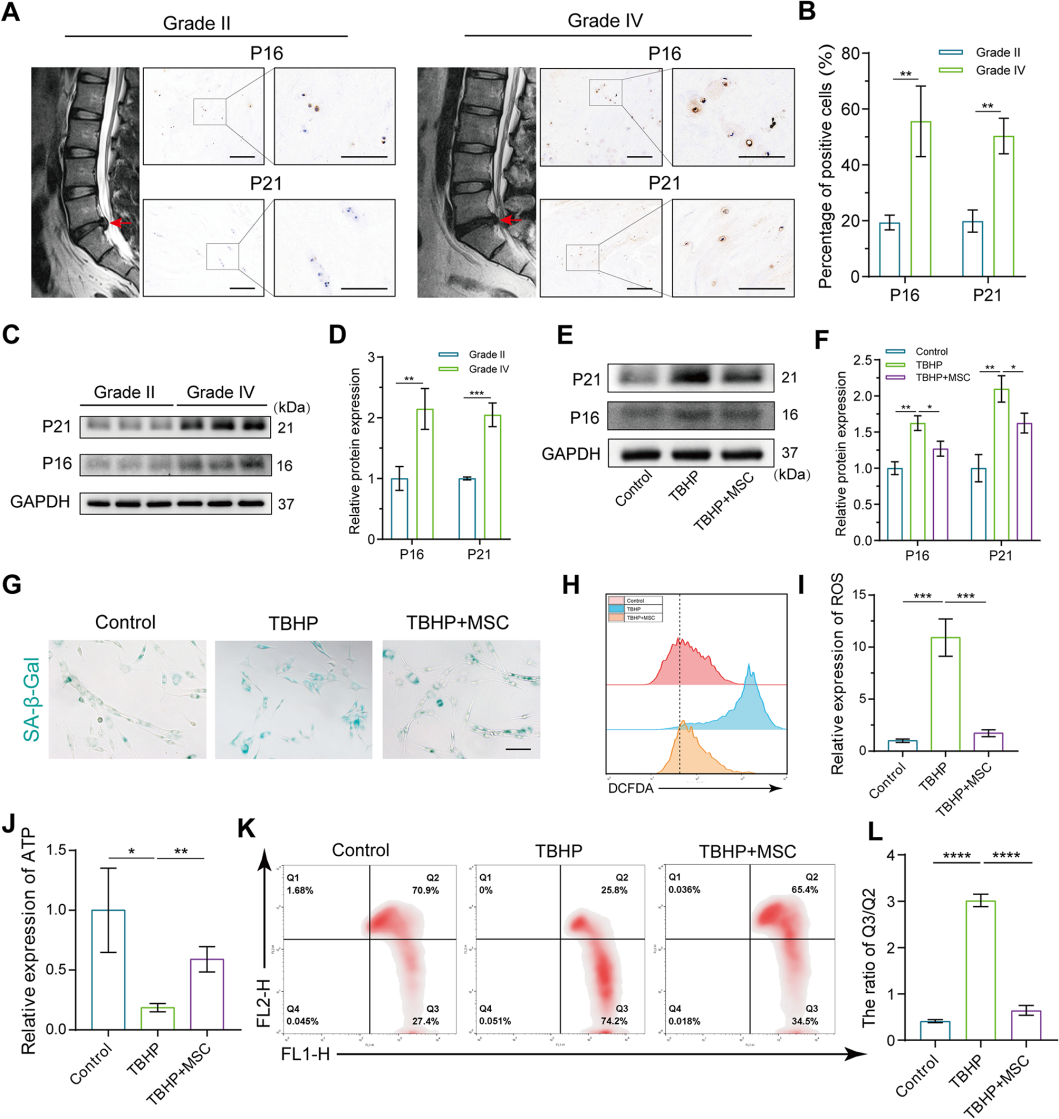
MSCs co-culture alleviated senescence of NPCs and mitochondrial dysfunction. (**A**) Representative MRI images and immunohistochemistry of senescence marker P16, P21 of HNP tissues with different degenerative degrees. (Scale bar: 100 μm–50 μm) (**B**) The percentage of P16, P21 positive cells in HNP tissues in Fig. 1A. (**C** and **D**) Western blot analysis and quantification of the protein expression levels of P16 and P21 in HNP tissues with different degenerative degrees. (**E** and **F**) NPCs were pretreated with 100 µM TBHP for 12 h and followed by MSCs co-culture for 48 h in transwell system. The expression of P16 and P21 was analyzed by western blot and image J. (**G**) Representative SA-β-Gal activity staining images of NPCs. NPCs were pretreated with 100 µM TBHP for 12 h and followed by coculturing with MSCs for 48 h in transwell system. (Scale bar: 100 μm) (**H** and **I**) The level of ROS and quantification of NPCs after the interventions in Fig. 1G. (**J**) The quantification of ATP generation of NPCs after the interventions in Fig. 1G. (**K** and **L**) The change of mitochondrial membrane potential and quantification of NPCs after the interventions in Fig. 1G. Data are represented as mean ± SD. *p < 0.05, **p < 0.01, ***p < 0.001, ****p < 0.0001

Current research posits that the diminishing activity and functionality of NPCs are primary factors in IVDD. In degenerated IVD, the pronounced senescence of NPCs significantly impacts the ECM secretion function, resulting in a locally compromised microenvironment that intensifies the onset of IVDD [3]. Surgical specimens from IVDs at varying degeneration levels were initially acquired, and senescence-related markers P21 and P16 were analyzed in degenerated disc tissues using immunohistochemical. As depicted in Fig. 1A and B, the expression of senescence-related markers in Pfirrmann Grade IV NP tissue is markedly higher than that in Grade II. This was further substantiated by WB, indicating increased NPC senescence with advancing IVDD (Fig. 1C and D).

As pivotal players in tissue engineering, MSCs have robust repair capabilities, and their therapeutic efficacy in degenerative diseases is well-established [33, 34]. By inducing NPC senescence with 100 µM TBHP in vitro and subsequent indirect co-culture of senescent NPCs with MSCs using the transwell system, we observed that MSCs effectively mitigated the TBHP-induced expression of senescence markers P21 and P16 (Fig. 1E and F). Additionally, the amelioration of NPC senescence was further affirmed through SA-β-Gal staining in MSCs co-culture (Fig. 1G). Cellular senescence commonly accompanies mitochondrial dysfunction, with excessive intracellular reactive oxygen species (ROS) disrupting mitochondrial energy supply and intensifying cellular senescence [35]. Pre-treatment of NPCs with TBHP induced an excess production of ROS and significantly inhibited ATP generation in NPCs. TBHP also notably induced depolarization of the mitochondrial membrane potential (MMP) in NPCs (Fig. 1H-L). However, MSCs co-culture partially rescued the excessive accumulation of ROS, ATP production, and MMP depolarization in senescent NPCs. These findings underscore that indirect MSCs co-culture can partially restore mitochondrial functionality in senescent NPCs.

### MSC mitigates NPC senescence by delivering mitoMVs

**Fig. 2:**
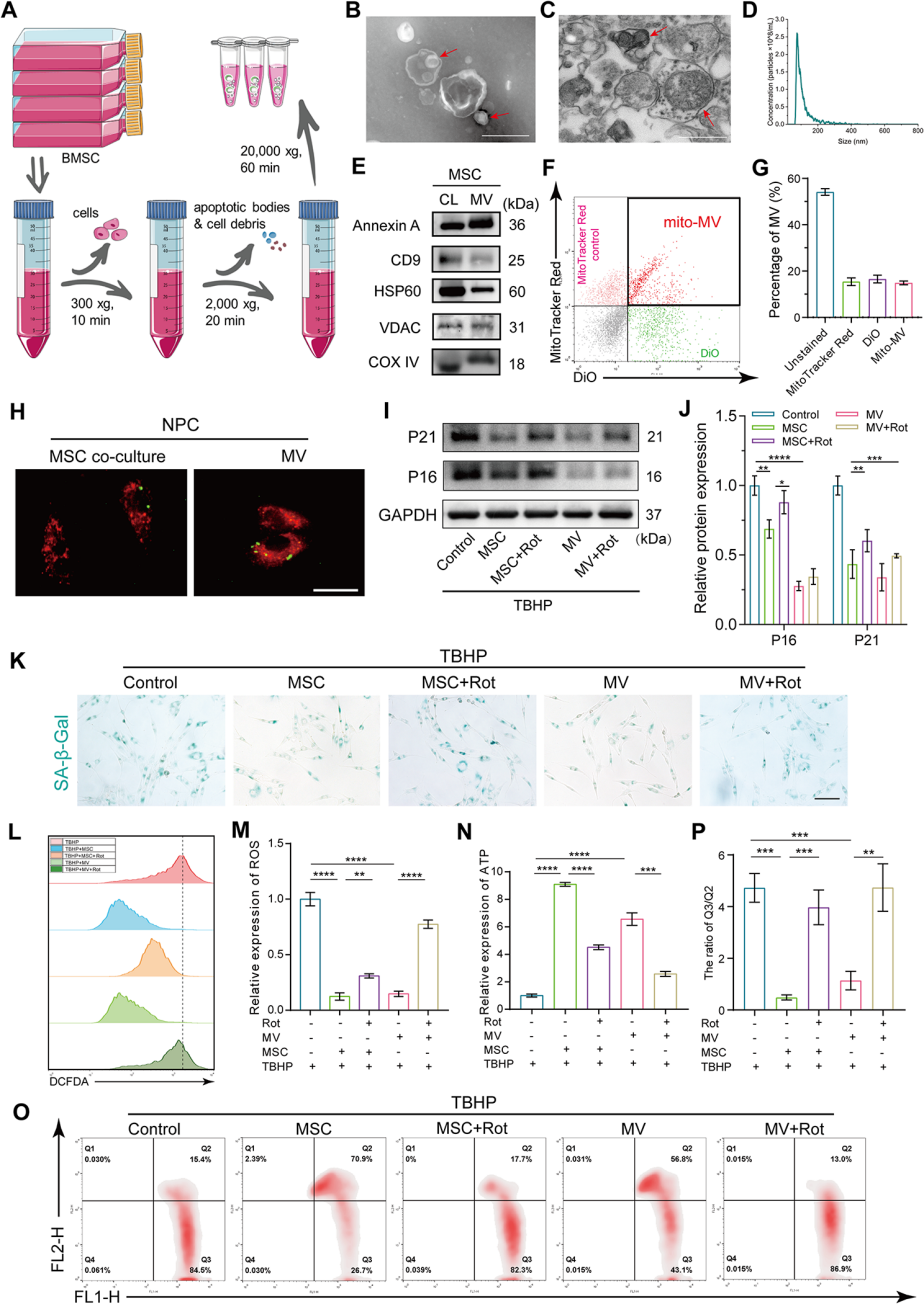
MitoMVs released by MSCs alleviated NPCs senescence and mitochondrial dysfunction. (**A**) MVs isolation schema. The supernatant of MSCs were collected and centrifuged according sequential centrifugation methods to get MVs. (**B**) TEM assay demonstrated negative-stain morphology images of MVs and its contains. (Scale bar: 500 nm) (**C**) TEM assay demonstrated cross-sectioned images of MVs and its contains after resin embedding. Mitochondria containing mitochondrial cristae structures can be clearly seen in the MVs. (Scale bar: 500 nm) (**D**) NTA demonstrated the concentration and size of MVs. (**E**) The protein marker of MVs (CD9, Annexin A) and proteins from the inner mitochondrial membrane, outer membrane, and mitochondrial matrix (HSP60, VDAC and COX IV) were analyzed by western blot. MSC whole cell lysate was used as a control. (**F**) MSCs were labeled with Mito-Tracker Red (red) and DiO (green), and MVs were isolated from MSCs supernatant and analyzed by flow cytometry. The Mito-Tracker Red single positive was marked as free mitochondria, the DiO single positive was labeled as MVs, and the Mito-Tracker Red-DiO double positive were mitochondria-containing MVs. (**G**) The quantification analysis of different subsets of MVs labeled with different dyes. (**H**) Representative fluorescence images of mitochondrial transfer in transwell culture system. NPCs labeled with mito-Tracker Red CMXRos (red) in the lower chambers were co-culture with mito-Tracker Green-labeled MSCs (green) from the upper inserts or isolated MVs (1 × 10^9/mL). (Scale bar: 50 μm) (**I** and **J**) NPCs seeded in the lower chambers were treated with 100 µM TBHP for 12 h and followed co-culture for 48 h with MSCs from the upper inserts or isolated MVs (1 × 10^9/mL), or pretreated MSC or MVs with 1 µM rotenone. The expression of senescence markers (P16 and P21) was analyzed by western blot and image J. (**K**) Representative SA-β-Gal activity staining images. NPCs seeded in the lower chambers were treated with 100 µM TBHP for 12 h and followed co-culture for 48 h with MSCs from the upper inserts or isolated MVs, or pretreated MSC or MVs (1 × 10^9/mL) with 1 µM rotenone. (Scale bar: 100 μm) (**L** and **M**) The level of ROS and quantification of NPCs after the interventions in Fig. 2K. (**N**) The quantification of ATP generation of NPCs after the interventions in Fig. 2K. (**O** and **P**) The change of mitochondrial membrane potential and quantification of NPCs after the interventions in Fig. 2K. Data are represented as mean ± SD. *p < 0.05, **p < 0.01, ***p < 0.001, ****p < 0.0001

In the therapeutic landscape of degenerative diseases, the biological activity of EVs released by MSCs stands out, complementing their intrinsic differentiation capabilities. These vesicles transport various cargoes that, upon internalization by recipient cells, orchestrate their fate. Several studies highlight the capacity of MSCs to rectify mitochondrial dysfunction in recipient cells through the release of mitochondria-containing MVs. To delve into whether MSCs intervene in mitochondrial dysfunction in NPCs and impede senescence by delivering mitochondria-containing MVs, we meticulously isolated MVs from the cell culture supernatant following the procedural outline depicted in Fig. 2A. Subsequent assessments of MV morphology, size, and concentration were conducted using transmission electron microscopy (TEM) and nanoflow cytometry. As depicted in Fig. 2B-D, the morphological resemblance of MVs to exosomes was evident, presenting elliptical shapes with distinctly visible circular contents. Further observation through TEM post-resin embedding disclosed elliptical contents within MVs exhibiting structures akin to mitochondrial cristae. Nanoflow cytometry unveiled an average size of 141 nm and a concentration of approximately 3 × 10^8/ml for the MVs. To affirm the presence of mitochondria in these vesicles, WB was employed to detect vesicle-associated markers and mitochondria-related proteins. The MV-associated markers CD9 and Annexin A confirmed the identity of the isolated vesicles as MVs. Additionally, the detection of mitochondrial inner membrane protein COX IV, matrix protein HSP60, and outer membrane protein VDAC within the vesicles confirmed the presence of mitochondria (Fig. 2E). A nuanced exploration into the proportion of MVs harboring mitochondria involved DiO and mito-Tracker Red CMXRos labeling of MSCs. Flow cytometric analysis discerned that double-positive MVs constituted approximately 20%, representing EVs encapsulating mitochondria (Fig. 2F and G). This result confirmed the release of mitochondria-containing MVs by MSCs.

Subsequent investigations focused on the prospect of MSCs delivering mitochondria to NPCs through MVs in an indirect co-culture setting. MSCs and NPCs were labeled with mito-Tracker Red CMXRos and mito-Tracker Green, respectively, and co-cultured within a transwell system. The results showed that mito-Tracker Red CMXRos-labeled MSC mitochondria seamlessly translocated to NPCs, integrating into the NPC mitochondrial network (Fig. 2H). Correspondingly, the introduction of MVs labeled with mito-Tracker Red CMXRos into the NPC culture medium revealed MSC-derived mitochondria within the NPC mitochondrial network (Fig. 2H). Consequently, the pivotal query probed whether MSCs secretion of mitochondria-containing MVs could effectively counteract NPC senescence and mitigate mitochondrial dysfunction. As delineated in Fig. 2I-P, co-culturing with MSCs or isolated MVs partially rescued NPC senescence and mitochondrial dysfunction. Intriguingly, pre-treatment with 1 µM rotenone (an inhibitor of mitochondrial complex I) reduced the therapeutic efficacy of delaying NPC senescence after MSC or MV co-culture. This underscored the pivotal role of mitoMVs released by MSCs in the amelioration of NPC senescence. Notably, rotenone pre-treatment also diminished the protective effect on mitochondrial function in senescent NPCs.

### SMFs enhances mitoMV secretion

**Fig. 3:**
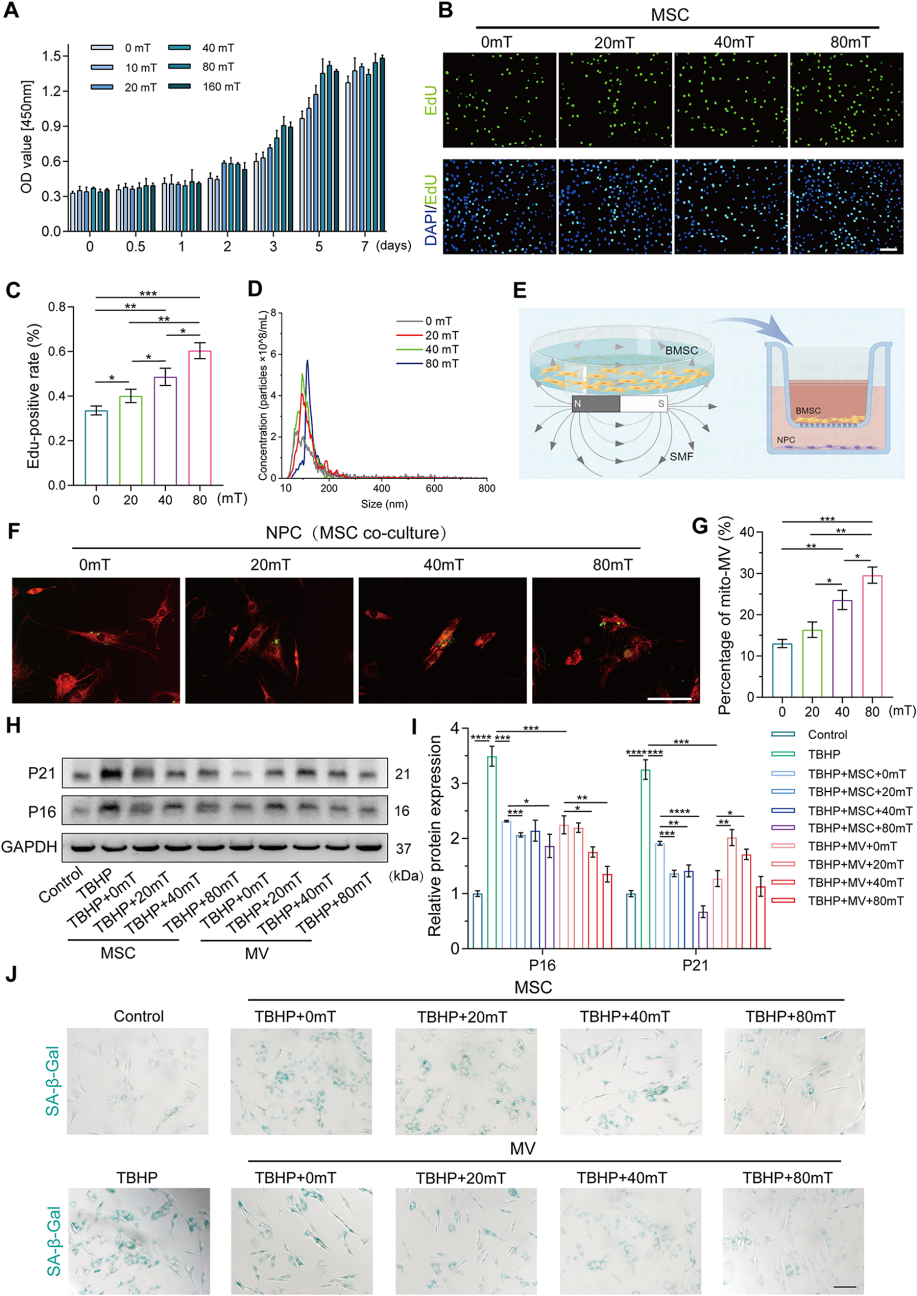
SMF enhances mitoMVs secretion and further alleviates NPCs senescence. (**A**) CCK-8 assay was used to detect the cell viability of MSCs after culturing with different magnetic field strengths. 20–80 mT and 72 h was selected as the follow-up intervention condition.(**B** and **C**) Representative images and quantification of EdU assay. The proliferative capacity of MSCs after different magnetic fields pretreatment was analyzed by EdU assay. (Scale bar: 50 μm) (**D**) NTA demonstrated the concentration and size of MVs secreted after intervention with different magnetic field strengths. (**E**) The schema of MSCs co-cultured with NPCs in transwell system. MSCs pretreatment with SMF were digested and seeded into the upper inserts, and then co-cultured with NPCs seeded in the lower chambers. This schema was created by Biorender. (**F**) Representative images of mitochondrial transfer in transwell culture system. NPCs labeled with mito-Tracker Red CMXRos (red) in the lower chambers were co-culture with mito-Tracker Green-labeled MSCs (green) after different magnetic fields pretreatment from the upper inserts. (Scale bar: 50 μm) (**G**) The quantification analysis of mitoMVs. The mito-Tracker Red and DiO-labeled MSCs were cultured in the different magnetic fields, and the MVs were isolated from MSCs supernatant and assessed the percentage of mitoMVs by flow cytometry. (**H** and **I**) NPCs seeded in the lower chambers were pretreated with 100 µM TBHP and followed co-culture with MSCs from the upper inserts or isolated MVs (1 × 10^9/mL) after different magnetic fields pretreatment. The expression of senescence markers (P16 and P21) was analyzed by western blot and image J. (**J**) Representative SA-β-Gal activity staining images. NPCs seeded in the lower chambers were pretreated with 100 µM TBHP and followed co-culture with MSCs from the upper inserts or isolated MVs (1 × 10^9/mL) after different magnetic fields pretreatment. Then the SA-β-Gal staining was processed. (Scale bar: 100 μm) Data are represented as mean ± SD. *p < 0.05, **p < 0.01, ***p < 0.001, ****p < 0.0001

SMFs have been recognized for their influence on cellular activities. Several studies indicate that moderate-intensity magnetic fields (1 mT-1 T) can significantly enhance the proliferative and differentiation capacities of MSCs [25]. We evaluated the impact of SMFs of varying intensities on the cellular activity of MSCs and observed a pronounced enhancement in cell activity under SMF conditions (Fig. 3A). Subsequently, we selected 20, 40, 80 mT and 72 h as intervention intensities and time. As shown in Fig. 3B and C, EdU results demonstrated a significant improvement in MSC proliferation under SMF interventions ranging from 20 to 80 mT. We further investigated whether SMF intervention affected the secretion of MVs. Nanoflow cytometry results revealed that SMF augmented MV secretion. Interestingly, under SMF interventions ranging from 0 to 80 mT, MV secretion increased proportionally with the strengthening of the magnetic field, reaching nearly three times that of the untreated group at 80 mT, with a secretion quantity of up to 6 × 10^8/ml (Fig. 3D). We co-cultured SMF-preconditioned MSCs with senescent NPCs and further assessed the impact of MSCs on senescent NPCs. As illustrated in Fig. 3E and F, SMF-preconditioned MSCs exhibited an enhanced capacity to deliver mitochondria, with more mitochondria from MSCs integrating into the mitochondrial network of senescent NPCs. Furthermore, we explored whether SMF enhanced the secretion of mitoMVs. Analysis of the conditioned media collected from SMF-preconditioned MSCs using flow cytometry revealed an increasing trend in the proportion of mitoMVs with the enhancement of SMF (Fig. 3G). These results confirm that SMF intervention not only increases the overall secretion of MSC-derived MVs but also enhances the secretion of mitoMVs. Subsequently, we investigated whether SMF-preconditioned MSCs alleviated NPC senescence. Co-incubation of senescent NPCs with SMF-preconditioned MSCs or SMF-preconditioned MVs significantly reduced senescence-related proteins and SA-β-Gal activity.

To further clarify the effects of MSC-derived MVs and exosomes on senescent NPCs after SMF preconditioning, we used co-culture inserts with different pore sizes to co-culture NPCs and MSCs. As shown in Fig. S1A-C, since the larger pores allow the passage of both MVs and exosomes, the alleviation of senescence was more pronounced with the larger pore inserts compared to the smaller pore inserts. Further, after 40 mT magnetic field intervention, we found that the SMF enhanced the anti-senescence effect. However, this effect was significant in the large pore inserts’ co-culture and not as evident in the small pore inserts’ co-culture.

To better distinguish the anti-senescence effects of MVs and exosomes, we separated MVs and exosomes from the conditioned medium of SMF-preconditioned MSCs using gradient centrifugation. Subsequently, we cultured senescent NPCs with either MVs or exosomes. We found that an equal amount of MVs was more effective in delaying NPC senescence than exosomes. Additionally, SMF-preconditioned MVs further enhanced their therapeutic effect, while exosomes did not show a significantly better therapeutic effect compared to non-SMF-preconditioned exosomes (Fig. S1D-I). In conclusion, SMF intervention primarily delays NPC senescence by enhancing MV secretion rather than exosome secretion.

### SMF enhances mitoMVs secretion by augmenting Kif5b-mediated mitochondrial transport

**Fig. 4:**
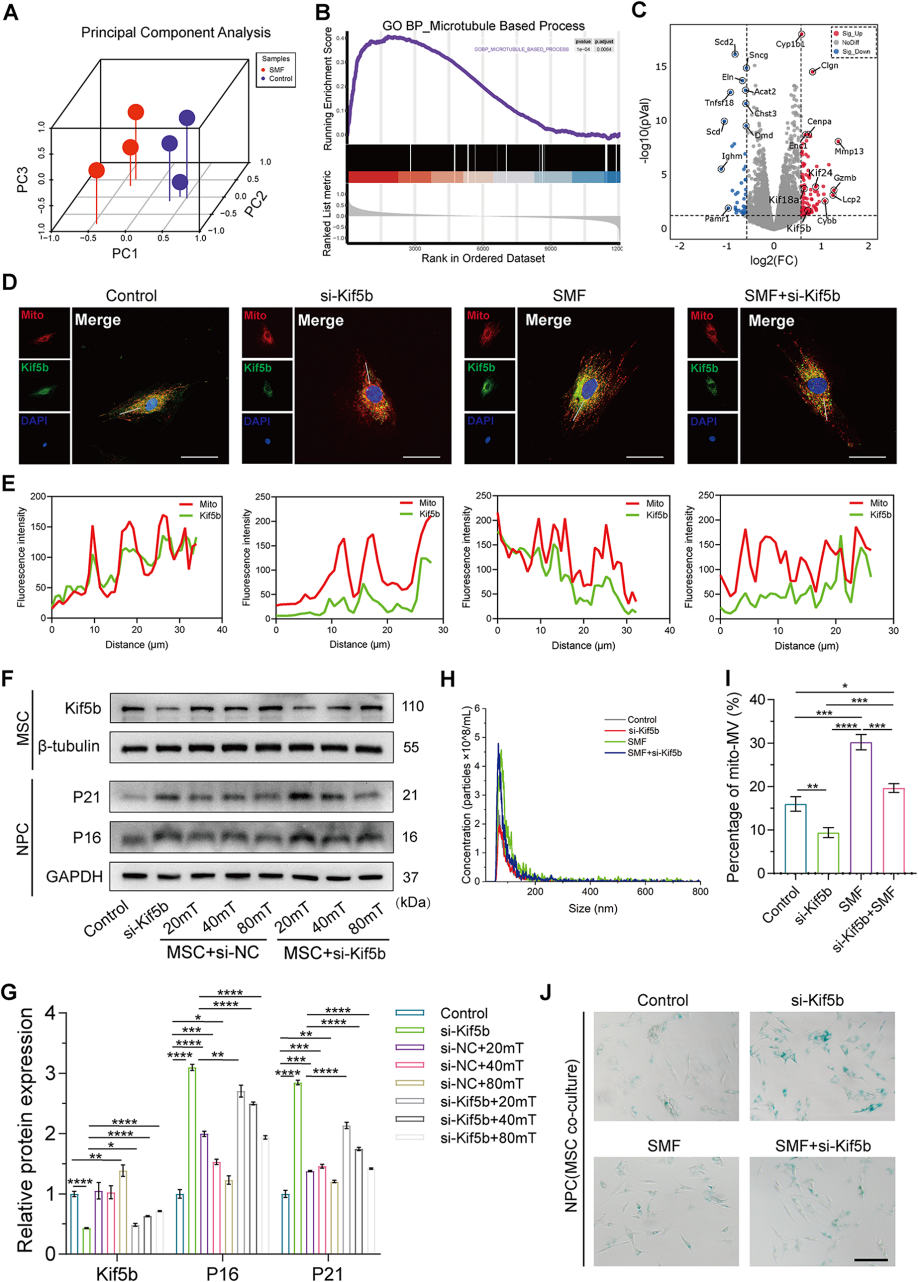
SMF enhances mitoMV secretion by enhancing mitochondrial transport by Kif5b in the cytoplasm. (**A**) Principal component analysis (PCA) of MSCs RNA sequencing data. (**B**) GSEA analysis of sequencing data demonstrated microtubule-based process of MSCs was enriched after treated with 40 mT SMF. (**C**) Volcano plot shows the differentially expressed genes of MSCs treated with SMF versus untreated. (**D** and **E**) Representative colocalization immunofluorescence images and quantification in MSCs. After transfection with si-Kif5b, MSCs were cultured with 40 mT SMF to assess colocalization of Kif5b (green) and mitochondrial (red). (Scale bar: 25 μm) (**F** and **G**) The level of protein expression and quantification of P16, P21 and Kif5b. After transfection with si-Kif5b or si-NC, MSCs were cultured with different magnetic fields and followed co-culture with NPCs pretreated with 100 µM TBHP for 48 h. (**H**) NTA demonstrated the concentration and size of MVs. After transfection with si-Kif5b, MSCs were cultured with 40 mT SMF for 72 h, and the MVs were isolated and analyzed from MSCs supernatant. (**I**) The quantification analysis of mitoMVs. After transfection with si-Kif5b, the mito-Tracker Red and DiO-labeled MSCs were cultured with 40 mT SMF for 72 h, and the MVs were isolated from MSCs supernatant and assessed the percentage of mitoMVs by flow cytometry. (**J**) Representative SA-β-Gal activity staining images of NPCs. After transfection with si-Kif5b, MSCs were cultured with 40 mT SMF for 72 h and followed co-culture with NPCs pretreated with 100 µM TBHP for 48 h. (Scale bar: 100 μm). Data are represented as mean ± SD. *p < 0.05, **p < 0.01, ***p < 0.001, ****p < 0.0001

To further elucidate the mechanism by which SMF enhances the secretion of mitoMVs, we conducted transcriptome sequencing (RNA-seq) on MSCs following 40 mT SMF intervention. Principal Component Analysis (PCA) results indicated a pronounced alteration in the transcriptional profile of MSCs after SMF intervention compared to the control (Fig. 4A). Further Gene Set Enrichment Analysis (GSEA) revealed enrichment of genes in multiple biological processes related to gene ontology, particularly those associated with microtubules (MTs) (Fig. 4B and Fig. S2A-D). Studies suggest that microtubules play a crucial role in the intracellular transport of various molecules, particularly in the redistribution and transport of mitochondria in axons. Through volcano plot visualization of significantly differentially expressed genes, as depicted in Fig. 4C, we observed a significant upregulation of the kinesin superfamily (KIF) genes. The kinesin family, acting as motor proteins, participates in the transport of intracellular cargo [36]. Therefore, we hypothesized that the kinesin family might be involved in the transport of mitochondria in the MSC cytoplasm. Prior research indicates that Kif5b is involved in the intracellular transport of mitochondria, especially in neurons, facilitating the supply of energy to cells [36].

To explore whether Kif5b participates in the transport of mitochondria in MSCs, we assessed the co-localization of Kif5b and mitochondria through immunofluorescence staining. As shown in Fig. 4D and E, Kif5b exhibited co-localization with mitochondria, and mitochondria seemed to be distributed systematically along the cytoskeleton throughout the cytoplasm. Furthermore, SMF intervention enhanced the co-localization of Kif5b and mitochondria, suggesting an increased capacity of Kif5b to transport mitochondria following SMF intervention. We silenced Kif5b in MSCs using siRNA and assessed mitochondrial distribution through immunofluorescence. The results revealed that mitochondrial distribution became disordered, primarily accumulating around the cell nucleus after Kif5b knockdown, while SMF intervention alleviated the disordered distribution of mitochondria post Kif5b knockdown. This indicates that Kif5b may participate in the vertical transport and transport processes of mitochondria in MSCs.

To validate the role of Kif5b in the intracellular transport of mitochondria, we subjected Kif5b-silenced MSCs to SMF intervention and co-cultured them with senescent NPCs. As shown in Fig. 4F and G, SMF intervention increased the protein expression level of Kif5b and rescued the protein levels affected by Kif5b knockdown. Moreover, SMF enhanced the therapeutic capacity of MSCs, as confirmed in Fig. 3. However, when Kif5b-silenced MSCs were co-cultured with senescent NPCs, their ability to alleviate NPC senescence significantly decreased. SMF treatment partially rescued the diminished ability of MSCs to alleviate senescence caused by Kif5b knockdown. We also investigated whether Kif5b affected MV secretion and the proportion of mitoMVs. As depicted in Fig. 4H and I, SMF intervention significantly increased MV secretion, whereas si-Kif5b treatment did not significantly affect MV secretion. These results suggest that Kif5b may not participate in the secretion process of MVs. We further assessed the proportion of mitoMVs after SMF intervention and si-Kif5b treatment. Interestingly, we found a significant increase in the proportion of mitoMVs following SMF intervention, while si-Kif5b affected the proportion of mitoMVs. These results suggest that Kif5b may influence mitochondrial transport, affecting the synthesis of mitoMVs, and does not play a regulatory role in MV secretion. When Kif5b was silenced, the therapeutic efficacy of MSCs in delaying NPC senescence diminished, while SMF intervention partially mitigated the reduced therapeutic capacity caused by si-Kif5b, confirming these conclusions through WB and SA-β-Gal assays (Fig. 4F and J).

### SMF enhances mitoMV secretion by regulating the interaction between Kif5b and Rab22a

Based on the aforementioned findings, we observed that Kif5b can influence the proportion of mitoMV through mitochondrial transport without affecting the overall MV secretion. This prompted us to delve further into the factors influencing mitoMV generation. The RAB family, a group of small GTPases, plays a pivotal role in regulating MV budding from the plasma membrane, inducing this process by contracting cortical actin beneath the membrane [37]. Within this family, Rab22a has been identified as a key molecule in regulating MV formation. Under hypoxic conditions, Rab22a mediates MV shedding in breast cancer cells, thus promoting breast cancer metastasis [38].

**Fig. 5:**
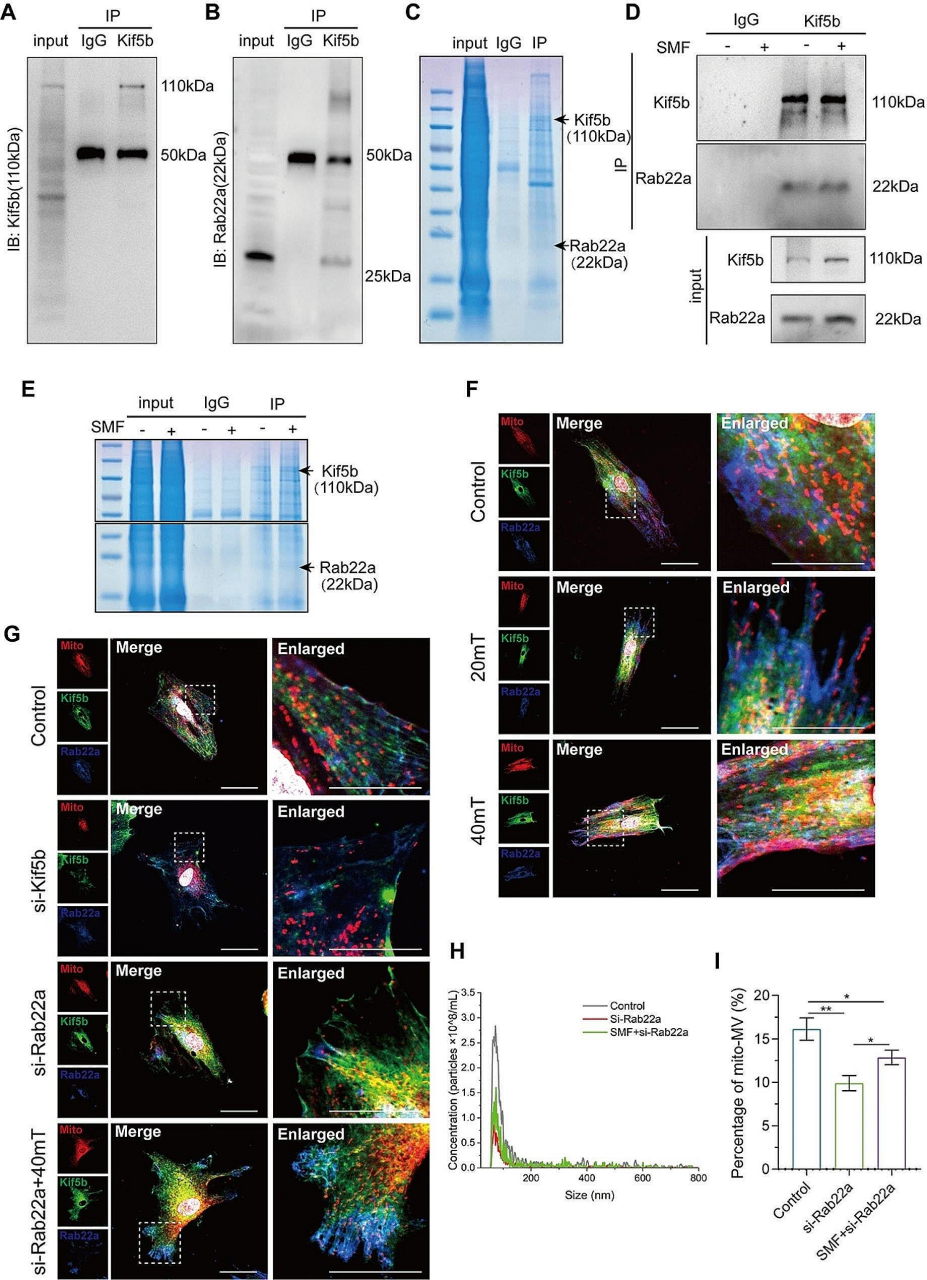
SMF enhances the interaction between Kif5b and Rab22a, and promotes the secretion of mitoMVs. (**A** and **B**) CO-IP assays were performed to measure the interactions between Kif5b and Rab22a in MSCs.Anti-Kif5b antibodies were used for CO-IP. (**C**) The coimmunoprecipitated protein was stained by Coomassie blue to demonstrates protein interaction between Kif5b and Rab22a. (**D**) CO-IP assays were performed to measure the change of interactions between Kif5b and Rab22a in MSCs pretreated with SMF. MSCs were cultured with 40 mT SMF for 72 h, and anti-Kif5b antibodies were used for CO-IP. (**E**) The coimmunoprecipitated protein was stained by Coomassie blue to demonstrates the change of interactions between Kif5b and Rab22a in MSCs pretreated with 40 mT SMF for 72 h. (**F**) Representative immunofluorescence images of colocalization of mitochondrial, Kif5b and Rab22a in MSCs. MSCs were pre-cultured with different magnetic field strengths for 72 h, and confocal microscopy was used to analyze the colocalization of mitochondrial (red), Kif5b (green) and Rab22a (blue). (Scale bar: 25 μm–10 μm) (**G**) Representative immunofluorescence images of colocalization of mitochondrial, Kif5b and Rab22a in MSCs. After transfection with si-Kif5b or si-Rab22a, MSCs were cultured with 40 mT SMF for 72 h, and confocal microscopy was used to analyze the colocalization of mitochondrial (red), Kif5b (green) and Rab22a (blue). (Scale bar: 25 μm–10 μm) (**H)** NTA demonstrated the concentration and size of MVs. After transfection with si-Rab22a, MSCs were cultured with 40 mT SMF for 72 h and the MVs were isolated and analyzed from MSCs supernatant. (**I**) The quantification analysis of mitoMVs. After transfection with si-Rab22a, the mito-Tracker Red and DiO-labeled MSCs were cultured with 40 mT SMF for 72 h, and the MVs were isolated from MSCs supernatant and assessed the percentage of mitoMVs by flow cytometry. Data are represented as mean ± SD. *p < 0.05, **p < 0.01, ***p < 0.001, ****p < 0.0001

Our hypothesis posits that Kif5b transports mitochondria to the plasma membrane, interacts with Rab22a, and triggers membrane budding mediated by Rab22a, culminating in the secretion of mitoMV. To validate this hypothesis, we initially assessed the interaction between Kif5b and Rab22a using CO-IP. As illustrated in Fig. 5A-C, a protein interaction between Kif5b and Rab22a was evident, further substantiated by Coomassie brilliant blue staining. To confirm that SMF intervention enhances the interaction between Kif5b and Rab22a, thereby promoting mitoMV secretion, CO-IP experiments were conducted. As shown in Fig. 5D, following SMF intervention, the protein expression levels of Kif5b and Rab22a in the input group were higher than those in the non-intervention group. In the IP group, SMF intervention did not significantly elevate Kif5b expression but seemed to enhance Rab22a protein levels compared to the non-intervention group. This suggests that SMF intervention amplifies the interaction between Kif5b and Rab22a, a finding corroborated by Coomassie brilliant blue staining (Fig. 5E). Immunofluorescence results affirmed that SMF treatment enhances the co-localization of mitochondria, Kif5b, and Rab22a in MSC (Fig. 5F).

To further substantiate the regulatory role of Rab22a in mitoMV secretion, siRNA was used to downregulate Rab22a expression. As depicted in Fig. 5G, Rab22a knockdown weakened their co-localization, albeit without apparent impact on the co-localization between Kif5b and mitochondria. Knocking down Kif5b, however, diminished the co-localization between Rab22a and mitochondria, indicating that Kif5b plays a pivotal bridging role in transporting mitochondria to Rab22a. Notably, SMF intervention partially alleviated this effect. Evaluation of whether Rab22a knockdown affects MV secretion and the proportion of mitoMV secretion revealed that si-Rab22a not only inhibited total MV secretion but also reduced the proportion of mitoMV (Fig. 5H and I). In conclusion, Rab22a emerges as a crucial player in regulating mitoMV secretion and represents a key factor in responding to SMF-mediated modulation of mitoMV secretion.

### Constructing a GelMA hydrogel system loaded with mitoMV

**Fig. 6:**
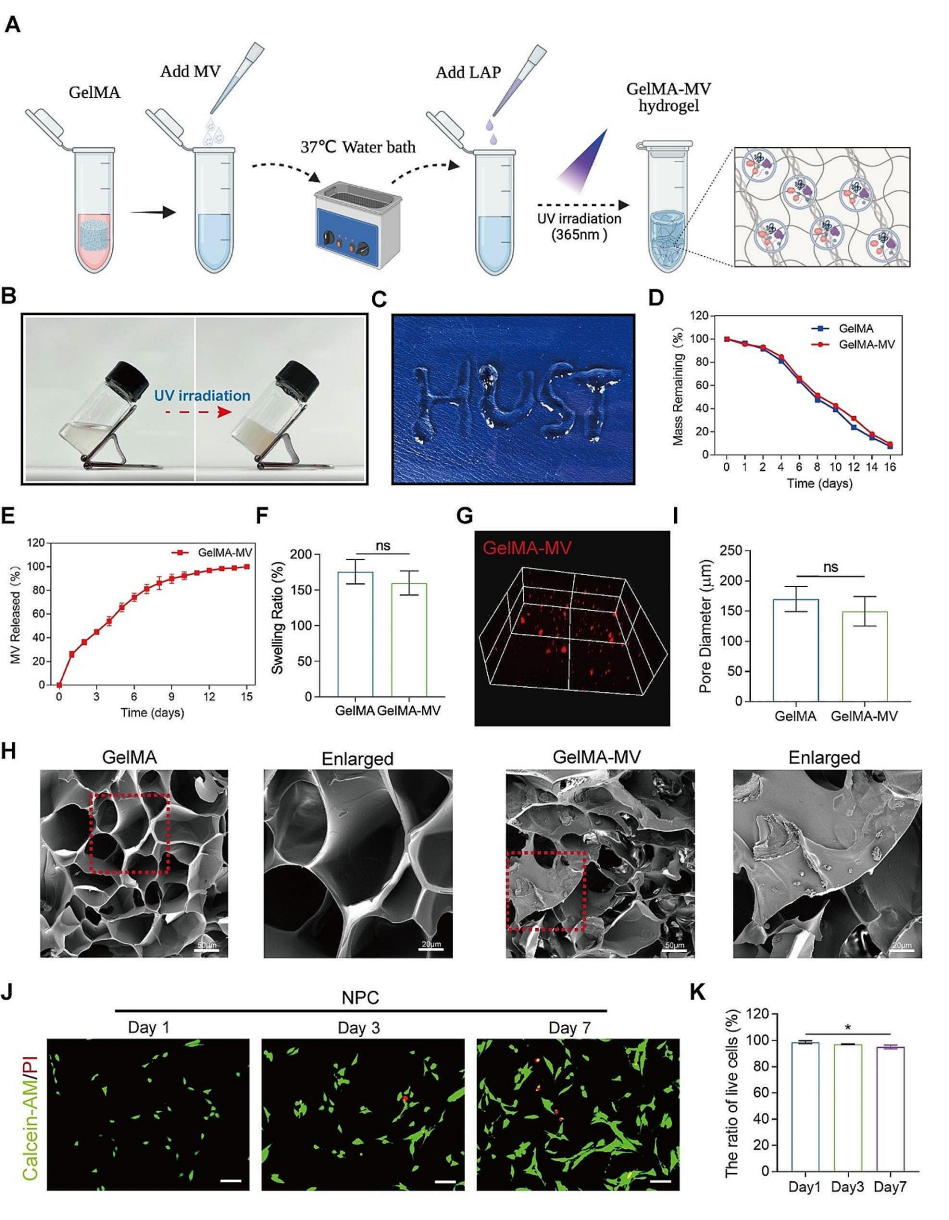
Synthesis and characterization of GelMA-MVs hydrogel sustained-release systems. (**A**) Schematic diagram of the synthesis process of the GelMA-MVs hydrogel sustained-release system. This figure was created by biorender. (**B**) Fluidable GelMA hydrogel transformed into concrete hydrogel via 365 nm photo- crosslinking. (**C**) The injectable GelMA hydrogel demonstrates the ability to vary the shape. (**D**) The degradation properties of GelMA and GelMA-MVs hydrogel in vitro. (**E**) GelMA-MVs hydrogels demonstrate the retardation property of MVs release in vitro. (**F**) The swelling properties of GelMA and GelMA-MVs hydrogel in vitro. (**G**) Confocal microscope shows the spatial distribution of DiR-labeled MVs (red) in the GelMA hydrogel. (**H**) The GelMA and GelMA-MVs hydrogel possesses a sparse, porous structure and surface scattered MVs observed by SEM after Lyophilization treatment. (Scale bar: 50 μm–20 μm) (**I**) The pore diameter comparison of GelMA and GelMA-MVs hydrogel. (**J** and **K**) Live/dead assay and quantitative analysis demonstrated the biocompatibility of NPCs cultured with GelMA hydrogels for 1, 3, 7 days. (Scale bar: 100 μm) Data are represented as mean ± SD. *p < 0.05, **p < 0.01, ***p < 0.001, ****p < 0.0001

A single injection can lead to the rapid release of EVs, resulting in a gradual decline in therapeutic efficacy over time. To achieve a sustained therapeutic effect, we constructed a hydrogel system to load MVs for prolonged and controlled release. Initially, as illustrated in Fig. 6A, we synthesized a photocurable GelMA hydrogel and loaded MV into the GelMA hydrogel system, followed by characterization and assessment. Figure 6B and C demonstrate the photocuring properties and injectability of GelMA hydrogel. Under UV irradiation at a wavelength of 365 nm, GelMA hydrogel could solidify into a gel within 20 s, highlighting its excellent photocuring characteristics. Moreover, its injectability provided the possibility for in vivo injection. Further evaluation of the in vitro degradation and release characteristics of the GelMA-MV hydrogel system is shown in Fig. 6D. The degradation period of GelMA hydrogel reached two weeks, and the GelMA hydrogel loaded with MV exhibited a slightly slower degradation rate than the MV-free GelMA hydrogel, providing an optimal platform for MV release. The MV release curve from the hydrogel displayed a gradual degradation process, sustaining for approximately two weeks in vitro (Fig. 6E). GelMA and GelMA-MV showed no significant differences in the swelling ratio and pore size (Fig. 6F and I). Confocal microscopy revealed the uniform distribution of MV within the GelMA hydrogel (Fig. 6G). Scanning electron microscopy (SEM) detected the internal structure of the freeze-dried GelMA hydrogel, displaying the even distribution of MV on the surface and internally (Fig. 6H), suggesting a gradual release of MV with the degradation of GelMA hydrogel. To further assess the biocompatibility of the GelMA hydrogel loaded with MV, we co-cultured NPCs with GelMA hydrogel. Live/dead staining after one week of co-culture revealed no significant cell death in NPC, further confirming the biocompatibility of GelMA hydrogel (Fig. 6J and K).

### GelMA-MV hydrogel system delays IVDD in vivo

**Fig. 7:**
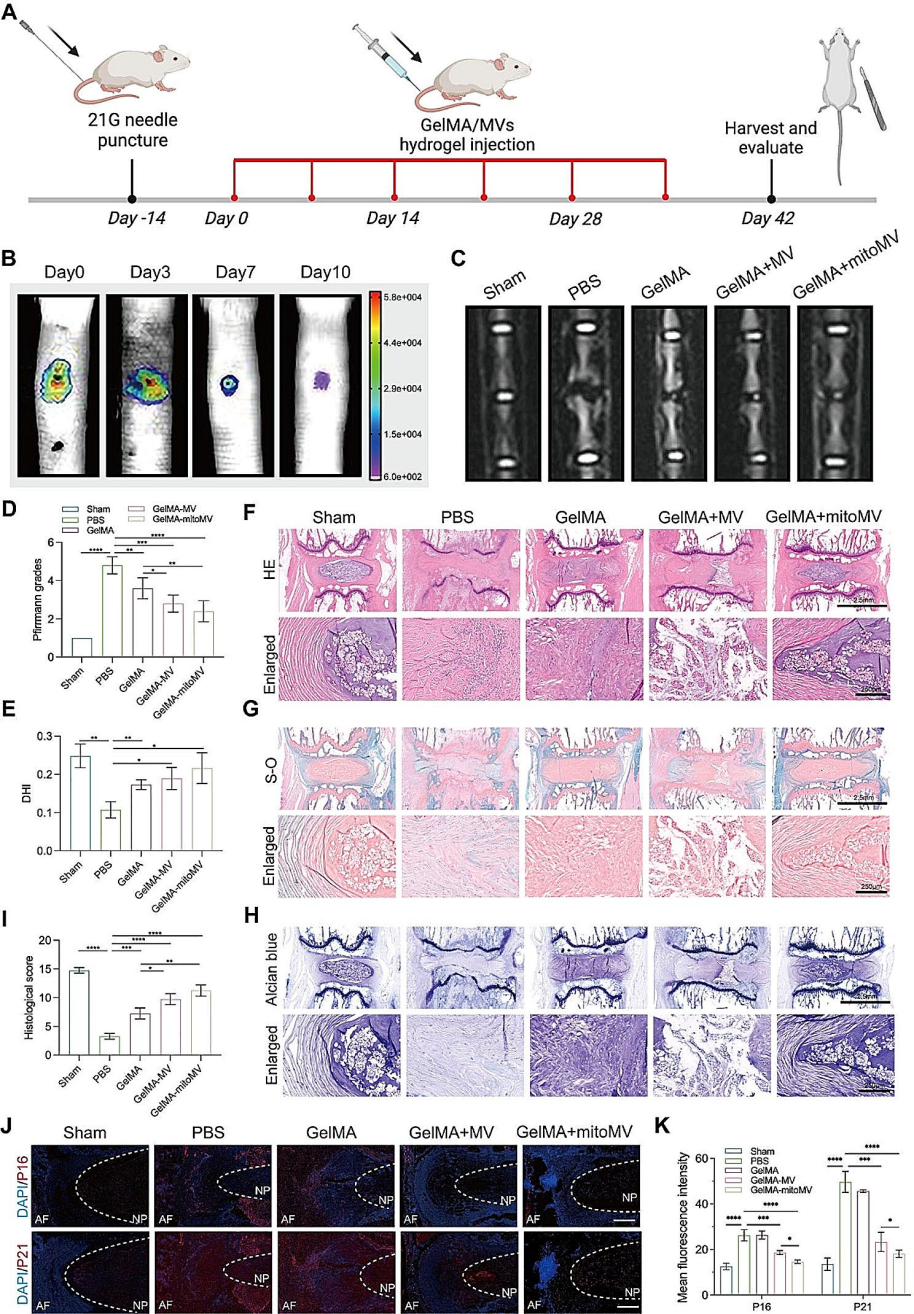
GelMA-MVs hydrogel sustained-release systems rescues IVDD in vivo. (**A**) Schematic diagram of the synthesis process of the GelMA-MVs hydrogel sustained-release system. This figure was created by biorender. (**B**) Representative fluorescence images of MVs in the IVD. DiR-labeled MVs (1 × 10^10/mL) were injected into IVD and in vivo imaging was performed at 0, 3, 7, 10 days, respectively. (**C**-**E**) Representative MRI images, quantification of Pfirrmann grades and DHI of IVD. (**F**-**H**) Representative images of H&E, Safranin fast-O green and alcian blue staining of IVD tissues. (Scale bar: 2.5–250 μm) (**I**) Quantification of histological grade of IVD tissues based on histological staining images. (**J** and **K**) Representative immunofluorescence images of senescence marker P16, P21 (Red) of IVD tissues and quantification analysis in different groups. (Scale bar: 250 μm) Data are represented as mean ± SD. *p < 0.05, **p < 0.01, ***p < 0.001, ****p < 0.0001

To validate the therapeutic efficacy of the GelMA-MV hydrogel system, we initially established an animal model of IVDD using fine needle puncture, followed by a series of imaging and histological assessments (Fig. 7A). Two weeks post-puncture, the sham group, PBS group, GelMA group, GelMA-MV group, and GelMA-mitoMV group were injected with PBS, GelMA hydrogel, GelMA-MV hydrogel, and GelMA-mitoMV hydrogel, respectively. As depicted in Fig. 7B, MV locally retained in the mouse tail IVD for approximately 10 days. Consequently, we performed weekly local injections of GelMA-MV hydrogel to maintain the biological effects of MV. After the 6-week intervention, we used MRI to assess the condition of IVDD. Results indicated significant imaging changes induced by fine needle puncture, with NP signals almost disappearing in the T2 phase. Local injection of GelMA hydrogel improved the condition of IVDD. Moreover, both the GelMA-mitoMV and GelMA-MV groups exhibited significantly enhanced NP signals, suggesting a marked improvement in IVDD. Furthermore, the therapeutic effect of the GelMA-mitoMV group after SMF intervention surpassed that of the GelMA group (Fig. 7C and D).

We further measured the intervertebral space height and found that the disc height index (DHI) in the PBS group was significantly lower than that in other intervention groups. The interventions with GelMA hydrogel, GelMA-MV hydrogel, and GelMA-mitoMV hydrogel significantly alleviated the loss of intervertebral space height (Fig. 7E). Histological analyses, including H&E, Picrosirius Red, and Alcian Blue staining, confirmed features of tissue degeneration, such as ECM loss, fibrosis, calcification, and indistinct boundaries between nucleus pulposus and annulus fibrosus tissues, induced by fine needle puncture. Treatment with GelMA-MV and GelMA-mitoMV led to regeneration of NP tissue and gradual restoration of ECM (Fig. 7F-I). To further assess the alleviation of IVD senescence by different interventions, we measured the expression of senescence markers P16 and P21 at the tissue level. Immunofluorescence results showed significant senescence changes in the intervertebral disc after puncture, while GelMA-MV and GelMA-mitoMV treatments significantly delayed IVD aging, with GelMA-mitoMV treatment showing the most pronounced alleviation in IVD aging (Fig. 7J and K). In conclusion, the above results confirm that the GelMA-MV hydrogel system has a notable effect in delaying IVDD in vivo, with the therapeutic effect of GelMA-mitoMV surpassing that of GelMA-MV and GelMA hydrogel alone.

## Discussion

IVDD is closely associated with the senescence of NPCs and a decline in cellular activity. Cell death within the degenerative IVD environment disrupts tissue homeostasis and triggering the release of pro-inflammatory cytokines. This cascade leads to a loss of proteoglycan content and composition, resulting in reduced disc hydration, height, and flexibility [39, 40]. IVDD further impairs the spinal ability to withstand physiologically acceptable loads during daily activities and affects the function of adjacent tissues, including muscles and ligaments [41]. Despite significant advances in IVDD treatments over the years, a definitive cure remains elusive. Conventional therapies, including surgery, non-steroidal anti-inflammatory drugs (NSAIDs), analgesics, muscle relaxants, and physical therapy, offer short-term symptom relief but fail to address the underlying pathology [5]. In recent years, regenerative medicine approaches have garnered increasing attention in the treatment of IVDD. Given the complex nature of IVD, regenerative strategies aim to stimulate the resident endogenous progenitor cell populations and supplement the IVD with new exogenous cells or cell derivatives to minimize cell death and the associated senescence-associated secretory phenotype (SASP) signaling cascades.

To promote endogenous repair of degenerated IVDs, stem cells derived from various sources have been transplanted into host tissues. Stem cells, with their differentiation properties and self-renewal capabilities, generate specific differentiated cells to replenish the cellular pool in targeted tissues [42]. Additionally, growth factors and cytokines secreted by stem cells play crucial roles in enhancing the activity of resident cells within the IVD and stimulating local tissue cells [43]. However, the tumorigenicity of pluripotent cells and the harsh microenvironment of the host IVD significantly hinder the therapeutic efficacy of stem cells in degenerated discs [44]. Current clinical trials involving MSCs for IVDD treatment have not demonstrated a definitive advantage of cell-based therapies for IVDD. As an alternative to stem cells, EVs play a pivotal role in maintaining intercellular communication. Inheriting specific proteins, lipids, and nucleic acids from their parent cells, EVs interact with target cells, influencing their behavior and phenotype [3]. Moreover, the low immunogenicity and adaptability of EVs to harsh conditions provide a favorable premise for their application in IVDD treatment. Depending on their biogenesis, size, and morphology, EVs are classified into exosomes (30–150 nm), MVs (200–1000 nm), and apoptotic bodies (50–2000 nm) [24]. Recent systematic reviews have confirmed that stem cell-derived EVs can retard the progression of IVDD at the molecular, cellular, and organ levels [45]. Additionally, ongoing clinical trials using platelet-derived EVs for IVDD treatment provide new evidence supporting EV therapy [5].

Mitochondrial dysfunction recognized as a key driver in various degenerative diseases [46, 47]. However, the comprehensive regulatory mechanisms governing mitochondrial function across cells or organs in both physiological and pathological states remain elusive, posing potential impediments to the advancement of mitochondrial-targeted therapies. This study convincingly demonstrates that, during the onset and progression of IVDD, NPCs undergo progressive senescence accompanied by mitochondrial dysfunction. Serving as pivotal seeding cells in tissue engineering, MSCs have garnered widespread validation for their therapeutic efficacy in degenerative diseases. Through co-culture experiments, we substantiate that the senescence phenotype and mitochondrial dysfunction in senescent NPCs can be ameliorated when co-cultured with MSCs. This necessitates further elucidation of the pivotal role played by EVs in mitigating the senescence process of NPCs.

Beyond the reparative action of MSCs through self-differentiation, the paracrine effect of transplanted MSCs, facilitated by EVs, emerges as a critical mediator in regulating cell activity by transferring nucleic acids and protein cargoes, thereby fostering intercellular communication [3]. Notably, MVs, characterized by larger size, can carry more diverse contents, and studies indicate that vesicular mitochondrial transport is mediated by MVs. In osteoblasts, MVs release mitochondrial fragments and mitochondrial-derived vesicles (MDVs), fostering a positive feedback loop that promotes osteogenic differentiation of bone progenitor cells and, consequently, bone formation [48]. We ascertain the collection of MVs through differential centrifugation and their characterization, affirming that MSCs can release MVs containing mitochondria. Further loss- and gain-of-function experiments underscore the pivotal role of mitochondrial-containing MVs in delaying NPC senescence and mitochondrial dysfunction.

The secretion of EVs is subject to modulation by the external environment, and under different conditions, variations in the quality and quantity of EVs are evident [25, 49]. A magnetic field’s ability to modulate cell activity, enhancing the secretion of EVs, hinges on altering cell membrane polarity [50, 51]. This study substantiates that SMF intervention significantly increases MV secretion and promotes the secretion of a subset of EVs containing mitochondria. However, the mechanisms underpinning how SMF enhances the occurrence of mitochondria-containing EVs and the transport of mitochondria to MVs remain elusive. Relative to exosomes, MVs differ in size, cargo, and formation mechanisms. The vertical transport of molecular cargoes to the plasma membrane is considered a prerequisite for the secretion of MVs containing specific cargoes [52]. Molecular cargo transport within cells primarily occurs through the microtubule and actin cytoskeleton transport proteins. Mitochondrial transport in neuronal cells predominantly relies on the microtubule motor protein transport system, consisting of kinesin superfamily proteins (KIF) and cytoplasmic dynein, Milton/Trak (adapters), and Miro (mitochondrial receptor proteins) [36, 53]. The Kif5 family of motor proteins, also known as Kif5, is the main motor that drives the anterograde transport of mitochondria towards the ends of neurons. Among the three subtypes of Kif5 in mammals (Kif5a, Kif5b, and Kif5c), Kif5b is widely expressed in somatic cells [36]. The N-terminal of Kif5 contains the motor domain of ATPase, while the C-terminal end is the cargo-binding domain, linking proteins that connect mitochondria. It has been confirmed that disrupting Kif5-mitochondrial coupling in hippocampal neurons impairs mitochondrial transport, reducing the density of mitochondria in the distal axon [54]. Disrupting Kif5b impairs mitochondrial transport and leads to perinuclear accumulation of mitochondria [55, 56]. This study, through co-localization experiments, establishes the involvement of Kif5b in the vertical transport of mitochondria within MSCs. Knocking down Kif5b using siRNA results in disordered mitochondrial distribution within the cytoplasm of MSCs and the occurrence of perinuclear aggregation. Furthermore, after knocking down Kif5b, the proportion of MVs containing mitochondria secreted by MSCs significantly decreases, and SMF pretreatment can rescue this phenomenon. This further confirms that Kif5b-mediated mitochondrial transport is a crucial prerequisite for driving the occurrence of mitoMV.

The primary mechanism governing the formation of MVs is plasma membrane budding, a process involving the redistribution of membrane lipids and the contraction mechanism of the cortical cytoskeleton [52]. Changes in the composition and architecture of membrane proteins and lipids are closely associated with calcium-dependent enzymes, flip-flop enzymes, and Ca^2+^-induced asymmetric membrane phospholipid rearrangements [23]. The regulation of MV detachment from the plasma membrane is intricately linked to the interaction of actin cytoskeleton proteins, including myosin and myosin-binding ATPase, with ATP-dependent contraction [57]. Active small GTPases of the Ras and Rho families play a crucial role in the vesicle transport process, especially in the ATP-dependent contraction induced by the interaction of actin cytoskeleton proteins [58, 59]. Rab proteins, membrane-bound GTPases, are vital for the formation, transport, and membrane fusion of vesicles. Rab22a has been reported to be closely associated with MV generation, with its overexpression leading to increased MV shedding [38]. Additionally, under hypoxic conditions, Rab22a may selectively recruit proteins to MVs and participate in the plasma membrane budding process. However, whether Rab22a is involved in the generation of MVs containing mitochondria remains to be explored. Our study confirms that SMF intervention significantly upregulates the expression of Rab22a and its interaction with Kif5b. This interaction allows Kif5b-loaded mitochondria to be delivered to Rab22a, subsequently encapsulated into budding MVs, thereby promoting the synthesis and secretion of mitoMV.

Hydrogels, characterized by high water content, excellent biocompatibility, and degradability, represent a three-dimensional network polymer mimicking the extracellular matrix, providing an ideal repair platform for degenerated IVD. Numerous studies have demonstrated that hydrogels are the most desirable biomaterial for promoting IVDD. GelMA hydrogel, in particular, has shown excellent therapeutic effects in anti-inflammatory, promoting ECM repair, and enhancing NPC activity. Researchers have designed an injectable light-crosslinked porous GelMA/silk fibroin methacrylate (SilMA) hydrogel, encapsulating MSCs. The results confirm that this hydrogel system promotes M2 polarization of macrophages for anti-inflammatory therapy, simultaneously delivering MSCs to facilitate cartilage repair [60]. Hydrogel-loaded exosomes have shown promising applications in various tissue regeneration fields, such as bone, cartilage, heart, nerve, and skin. Although there is ample evidence supporting the efficacy of EVs in treating IVDD, local injection of EVs into tissues can lead to their sudden release and inadequate stability, resulting in suboptimal therapeutic effects. To enhance the stability and sustained release of EVs within tissues, this study constructs a GelMA-MVs hydrogel release system. Animal experiments validate the excellent therapeutic efficacy of the GelMA-MVs hydrogel release system in delaying IVDD.

In summary, our study explores the molecular mechanisms by which SMF enhances MSC secretion of mitoMV, elucidates the crucial roles of Kif5b and Rab22a in mitochondrial transport and the promotion of mitoMV secretion. Additionally, we have constructed a mitoMV release delivery system, further applying it to the process of IVDD, offering novel insights for regenerative treatments of IVD.

## Conclusions

In conclusion, this study reveals that MSCs can mitigate NPC senescence by secreting mitoMV, and further confirms that SMF enhances the secretion of mitoMV by MSCs. Given the distinct generation mechanisms of MVs compared to exosomes, we focused the investigation into the molecular mechanisms by which SMF enhances mitoMV secretion on processes such as cargo transport and plasma membrane budding related to MV formation. Subsequent RNA sequencing provided additional evidence suggesting the involvement of microtubule-based transport protein Kif5b in the transport process of mitoMV after SMF intervention. We also verified that Rab22a, through interaction with Kif5b, facilitates the sorting of mitochondria into MVs and potentially mediates subsequent plasma membrane budding processes. Following this, we demonstrated the significant potential of mitoMV in delaying IVDD through the construction of a GelMA hydrogel delivery system, offering novel insights for future treatments of IVDD.

### Electronic supplementary material

Below is the link to the electronic supplementary material.

Supplementary Material 1

## Data Availability

The data that support this study are available from the corresponding author upon reasonable request.
