# Supplementary material for: Static magnetic field-modulated mesenchymal stem cell-derived mitochondria-containing microvesicles for enhanced intervertebral disc degeneration therapy
Source: J Nanobiotechnology. 2024 Jul 31;22:457. doi: 10.1186/s12951-024-02728-6 (PMC11290117; doi:10.1186/s12951-024-02728-6)
Supplement: Supplementary file 1 — Supplementary Material 1 [file 12951_2024_2728_MOESM1_ESM.docx]

**Supplementary material**

**Table S1.** The sequences of si-Kif5b and si-Rab22a.

| Name | Sequences |
| --- | --- |
| si-Kif5b _001 | GGAGCUAGUCCAACUUCGA |
| si-Kif5b _002 | GACACGUAGCAGUUACAAA |
| si-Kif5b _ 003 | GGAUCAAGAUAAUAUGCAA |
| si-Rab22a_001 | CCAUCAUCGUCUAUGACAUTT |
| si-Rab22a_002 | GGUUUGUGGAAGACAGCUUTT |
| si-Rab22a_003 | CCGAUUCCAUUCAUGCCAUTT |


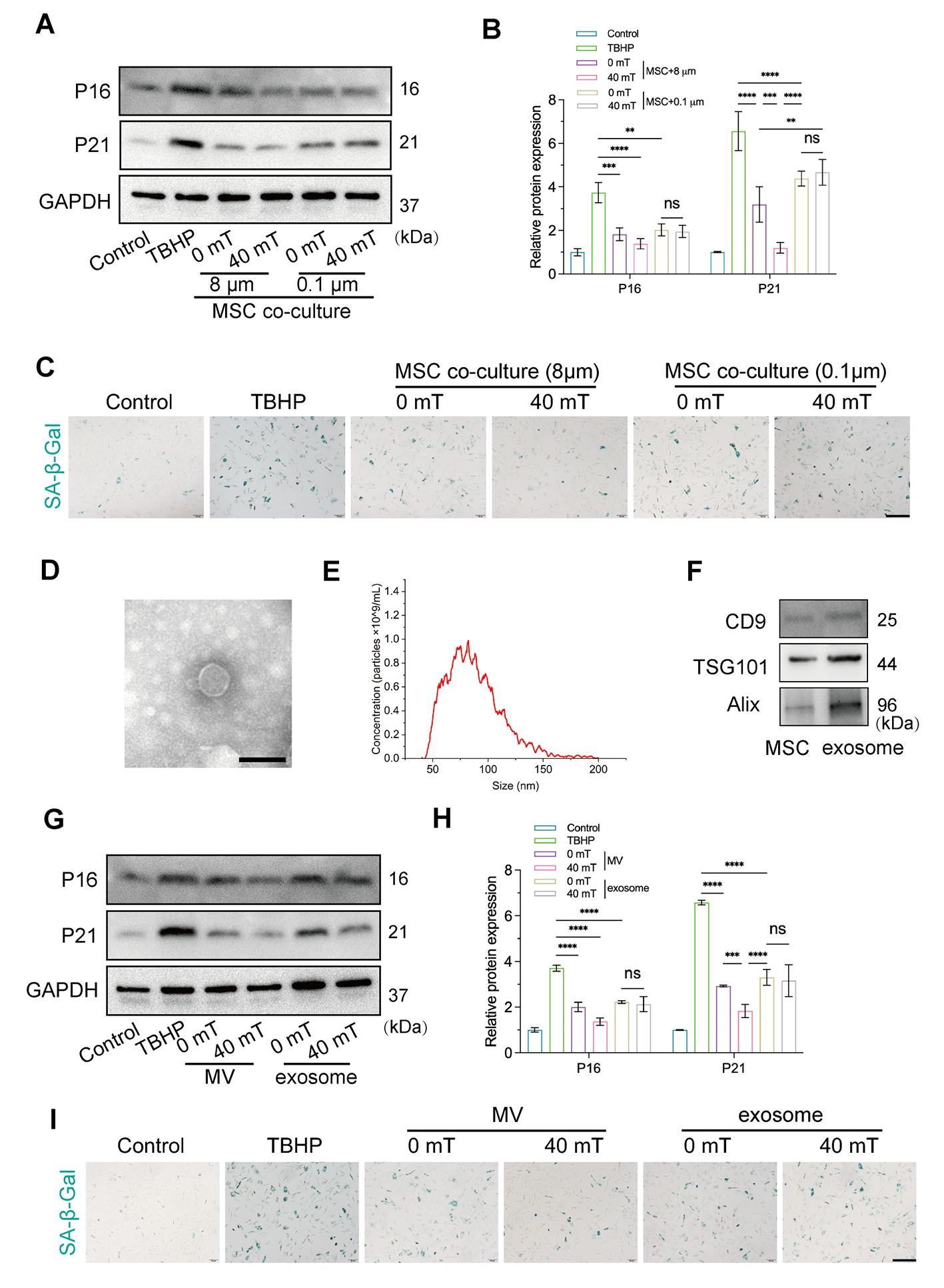


**Figure S1.** SMF enhances the anti-senescence effects of MVs rather than exosomes. (**A** and **B**) NPCs seeded in the lower chambers were pretreated with 100 μM TBHP for 12 h and followed co-culture for 48 h with MSCs from the upper inserts (pore sizes: 8 µm or 0.1 µm) after 40 mT magnetic fields pretreatment. The expression of senescence markers (P16 and P21) was analyzed by western blot and image J. (**C**) Representative SA-β-Gal activity staining images. NPCs seeded in the lower chambers were pretreated with 100 μM TBHP for 12 h and followed co-culture for 48 h with MSCs from the upper inserts (pore sizes: 8 µm or 0.1 µm) after 40 mT magnetic fields pretreatment. Then the SA-β-Gal staining was processed. (Scale bar: 200 μm) (**D**) TEM assay demonstrated negative-stain morphology images of exosome. (Scale bar: 100 nm) (**E**) NTA demonstrated the concentration and particle size of exosome. (**F**) The protein marker of exosome (CD9, TSG101 and Alix) were analyzed by western blot. MSC whole cell lysate was used as a control. (**G** and **H**) MSCs were cultured with 0 mT or 40 mT SMF for 72 h, and the MVs and exosome were isolated from MSCs supernatant by gradient centrifugation. Then, NPCs were pretreated with 100 μM TBHP for 12 h and followed co-culture with MVs and exosome (1×10^9/mL). The expression of senescence markers (P16 and P21) was analyzed by western blot and image J. (**I**) Representative SA-β-Gal activity staining images. MSCs were cultured with 0 mT or 40 mT SMF for 72 h, and the MVs and exosome were isolated from MSCs supernatant by gradient centrifugation. Then, NPCs were pretreated with 100 μM TBHP for 12 h and followed co-culture with MVs and exosome (1×10^9/mL). Then the SA-β-Gal staining was processed. (Scale bar: 200 μm) Data are represented as mean ± SD. *p < 0.05, **p < 0.01, ***p < 0.001, ****p < 0.0001.


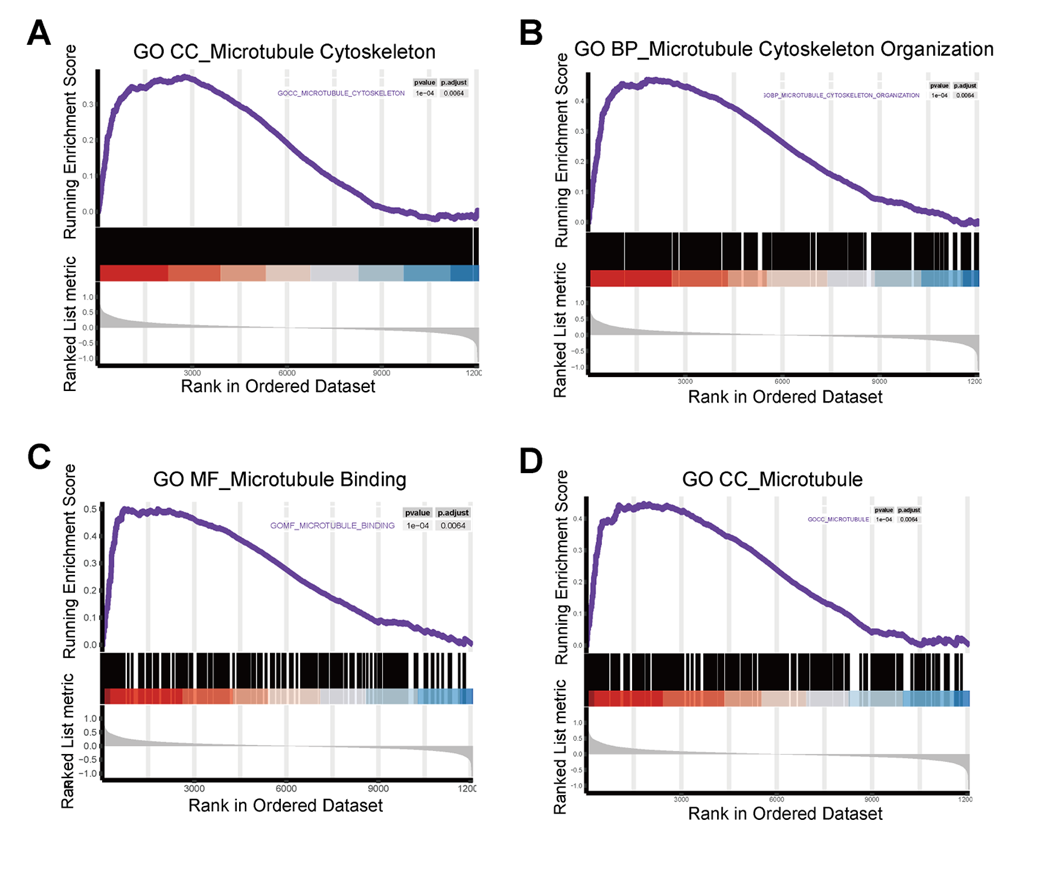


**Figure S2.** GSEA analysis demonstrates the enrichment of sequencing data in microtubule-related processes. (**A**) Sequencing data enriched in microtubule cytoskeleton of cellular component. (**B**) Sequencing data enriched in microtubule cytoskeleton organization of biological process. (**C**) Sequencing data enriched in microtubule binding of molecular function. (**D**) Sequencing data enriched in microtubule of cellular component.
